# State of the art and future challenges of urethra-sparing stereotactic body radiotherapy for prostate cancer: a systematic review of literature

**DOI:** 10.1007/s00345-023-04579-6

**Published:** 2023-09-05

**Authors:** Jennifer Le Guevelou, Davide Giovanni Bosetti, Francesco Castronovo, Antonio Angrisani, Renaud de Crevoisier, Thomas Zilli

**Affiliations:** 1 Radiation Oncology, Center Eugène Marquis, Rennes, France; 2 Department of Radiation Oncology, Oncology Institute of Southern Switzerland (IOSI), EOC, Via Ospedale, 6500 Bellinzona, Switzerland; 3 Facoltà Di Scienze Biomediche, Università Della Svizzera Italiana (USI), Lugano, Switzerland; 4 Faculty of Medicine, University of Geneva, Geneva, Switzerland

**Keywords:** Prostate cancer, SBRT, Radiotherapy, Hypofractionation, Urethra-sparing, Toxicity

## Abstract

**Purpose:**

Doses delivered to the urethra have been associated with an increased risk to develop long-term urinary toxicity in patients undergoing stereotactic body radiotherapy (SBRT) for prostate cancer (PCa). Aim of the present systematic review is to report on the role of urethra-sparing SBRT (US-SBRT) techniques for prostate cancer, with a focus on outcome and urinary toxicity.

**Method:**

A systematic review of the literature was performed on the PubMed database on May 2023. Based on the urethra-sparing technique, 13 studies were selected for the analysis and classified in the two following categories: “urethra-steering” SBRT (restriction of hotspots to the urethra) and “urethra dose-reduction” SBRT (dose reduction to urethra below the prescribed dose).

**Results:**

By limiting the urethra D_max_ to 90GyEQD2 (α/β = 3 Gy) with urethra-steering SBRT techniques, late genitourinary (GU) grade 2 toxicity remains mild, ranging between 12.1% and 14%. With dose-reduction strategies decreasing the urethral dose below 70 GyEQD2, the risk of late GU toxicity was further reduced (< 8% at 5 years), while maintaining biochemical relapse-free survival rates up to 93% at 5 years.

**Conclusion:**

US-SBRT techniques limiting maximum doses to urethra below a 90Gy_EQD2_ (α/β = 3 Gy) threshold result in a low rate of acute and late grade ≥ 2 GU toxicity. A better understanding of clinical factors and anatomical substructures involved in the development of GU toxicity, as well as the development and use of adapted dose constraints, is expected to further reduce the long-term GU toxicity of prostate cancer patients treated with SBRT.

## Introduction:

Radiation therapy (RT) represents one of the mainstay treatments for men diagnosed with localized prostate cancer (PCa) [1, 2]. The technological improvements of the past decade, with both implementation of intensity-modulated radiation therapy (IMRT) or rotational techniques [3, 4] and image-guided radiation therapy (IGRT) [5], have enabled the mitigation of genitourinary (GU) and gastrointestinal (GI) toxicity.

With a better understanding of the radiobiology of PCa [6], hypofractionation has become a new standard for the treatment of localized disease, including extreme hypofractionated schedules in 5 or fewer fractions delivered with stereotactic body radiation therapy (SBRT) [7–11]. Although toxicity results seem acceptable, further efforts to minimize long-term toxicities of SBRT are constantly explored. While GU toxicity after prostate SBRT appears multifactorial and associated with age, baseline urinary function, and prostate size, emerging data emphasize the role of SBRT doses delivered to urinary substructures.

In the recent literature, the urethra has been identified as a new organ at risk potentially influencing the long-term toxicity of patients treated with definitive radiotherapy, in analogy with data reported in brachytherapy (BT) series [12–15]. As doses delivered to urethra have been associated with the development of GU toxicity in SBRT studies, radiotherapy techniques aiming to optimize and reduce dose delivered at this level have been developed and implemented in several trials [16–18]. Nevertheless, urethra-sparing radiotherapy techniques suffers from a great variability, both with respect to the anatomical definition of the urethra as organ at risk for treatment planning and use of dedicated dose constraints for treatment optimization.

In order to shed light on the available evidence on this emerging topic, in the present study, we aim to present a systematic review of the literature regarding urethra-sparing SBRT (US-SBRT) techniques for PCa, with a focus on outcomes and urinary toxicity of two different urethra optimization approaches, the “urethra-steering” and the “urethra dose-reduction.”

## Material and methods

Eligibility criteria.

All trials reporting either toxicity or oncological outcomes after prostate SBRT were considered for inclusion. Studies were deemed eligible if they reported urethral dose-constraints either within their protocol or the manuscript. Studies were deemed to conduct urethra-sparing if they performed either “urethra-steering” (restriction of hotspots to the urethra) or “urethra dose-reduction” (maximal doses delivered to the urethra inferior to the dose of prescription to the prostate gland or dose-prescription on the urethra lower than dose of prescription to the target volume).

Information sources and search strategy.

A systematic search of the literature was performed in May 2023 on the PubMed database, using the MeSH term “urethra sparing.” Due to the scarcity of evidence on this topic, a broad search was voluntarily performed. There was no period restriction.

Selection process.

Two reviewers independently screened the articles, both at identification and screening process (J.L.G and T.Z.). Disagreements were discussed and resolved through consensus. For every study, the following data were retrieved: publication year, number of included patients, study design, radiation technique, dose delivered to the prostate gland, dose delivered to the urethra, median follow-up, toxicity outcomes (assessed by either RTOG or CTCAE grading scale), and oncological outcomes.

Synthesis method.

All studies meeting the inclusion criteria were selected for narrative synthesis. The results have been reported narratively, and summarized in tables when deemed appropriate. This systematic review was performed according to the Preferred Reporting Items for Systematic Reviews and Meta-Analyses guidelines [19].

## Results

### Study selection

**Fig. 1:**
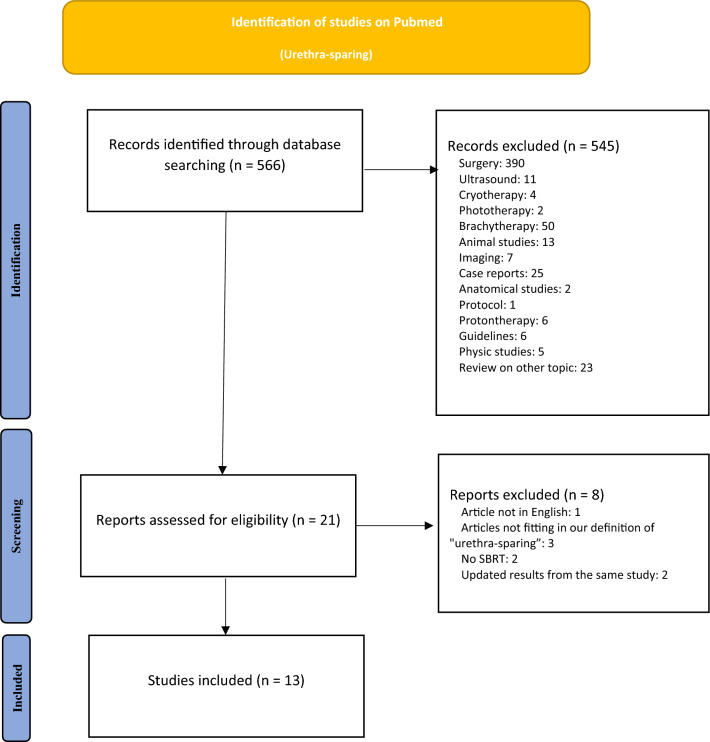
PRISMA flow chart

A flow chart of the literature screening is shown in Fig. 1. The search allowed to retrieve a total of 566 articles. After screening, 545 records that did not address the issue of urethra-sparing for PCa RT were excluded (surgical trials, brachytherapy trials, reviews…) leaving a total of 21 articles assessed for eligibility. After full-text reading, 8 additional reports were excluded (articles not in English, articles that did not fit our definition of urethra-sparing, treatment not performed using SBRT). A total of 13 articles were included in the present review.

## Urethra-steering SBRT

Most of trials performing urethra-steering implemented a whole gland prostate irradiation schedule with a simultaneous integrated boost (SIB) on the dominant intraprostatic lesion (DIL) (Table 1). McDonald et al*.* recruited 26 patients within a prospective pilot study to receive 36.25 Gy on the prostate gland with dose escalation up to 40 Gy to the DIL [20]. Within a 3-month follow-up, acute grade 2 toxicity occurred in 52% of patients, mostly consisting in dysuria and frequency. Two patients required catheterization for acute retention. Within the Hypo-FLAME trial, 35 Gy in 5 fractions was prescribed on the whole prostate gland, with dose escalation up to 50 Gy on the DIL [21]. While dose constraints on the urethra were limited to a D_max_ of 42 Gy as per protocol (equivalent to 96.6 Gy in standard fractionation using a α/β of 3 Gy for late toxicity – EQD2), the median delivered D_max_ reported in the study was 85.4 Gy EQD2. After a median follow-up of 18 months, acute and late grade 2 GU toxicity were reported in 34% and 14% of the patients, respectively. No patient experienced grade 3 toxicity. While patients received RT treatment once-weekly within the HYPO-FLAME trial, the same team assessed the safety of a reduction in overall treatment time from 29 to 15 days within the HYPOFLAME 2.0 trial [22]. Patients treated within the once-weekly arm experienced significantly less acute grade 2 GU toxicity than patients treated within the semi-weekly schedule (34% *vs* 47.5%, p = 0.01). No significant difference was observed with regard to acute grade 2 GI toxicity. Cloitre et al*.* led a phase I-II dose escalation trial with CyberKnife®, prescribing doses of 36.25 Gy in 5 fractions to the whole prostate, while simultaneously escalating doses to the DIL up to 50 Gy [23, 24]. Acute grade 2 GU toxicity was reported in 15% of men. Despite a toxicity flare observed 1 month after SBRT as assessed by the EORTC quality of life (QoL) PR-25 questionnaire and IPSS score, a return to the baseline status was observed at month 3. Late grade 2 GU toxicity was observed in 12.1% of the patients, consisting mostly in urinary frequency and urgency. No grade 3 GU toxicity occurred over the complete course of follow-up. These results were consistent with a median urethra delivered dose to 0.1 cc and 1 cc (D_0.1 cc_ and D_1cc_) of 83.5 Gy EQD2 and 77.7 Gy EQD2, respectively, lower than the maximal doses accepted as per protocol (91.8 Gy EQD2 and 84.2 Gy EQD2, respectively). Within a median follow-up of 61 months, 5-year biochemical relapse-free survival (bRFS) was 70%, including a 30% of intraprostatic (30%) relapse. Omission of androgen deprivation therapy (ADT) in the majority of intermediate- and high-risk patients may explain these results.

**Table 1:** Toxicity and oncological outcomes after urethra-steering protocols (limitation of maximal doses, with maximal doses delivered to the urethra lower than maximal doses delivered within the target volume)

Author	Patients	Study design	RT technique	RT dose	Urethral maximum dose (EQD2, α/β = 3 Gy)	ADT	Median follow-up	Toxicity scale	Acute GU toxicity	Acute GI toxicity	Late GU toxicity	Late GI toxicity	Oncological outcomes
Herrera et al., 2019 [24] Cloitre et al., 2023 [23]	33 pts IR = 42% HR = 55%	Phase I/II prospective trial	CyberKnife (82%) Tomotherapy	36.25 Gy / 5 fx Dose escalation up to 50 Gy / 5 fx to the DIL	D_1cc_ < 39 Gy (84.2 Gy _EQD2_) D_0.1 cc_ < 41 Gy (91.8 Gy _EQD2_)	3% (IR)	61 months	CTCAE	G2: 15%	G2: 6.1%	G2: 12.1%	G2: 3%	bRFS: 70%
Draulans et al*.,* 2020 [21] [22]	100 pts IR = 25% HR = 75%	Phase II prospective trial (HYPO-FLAME)	VMAT	35 Gy / 5fx Dose escalation to 50 Gy / 5 fx to the DIL	D_max_ < 42 Gy (96.6 Gy _EQD2_) D_max_ delivered (85.4 Gy _EQD2_)	31% short-term 31% long-term	18 months	CTCAE	Cumulative at 3 months: G2: 34% G ≥ 3: 0%	Cumulative at 3 months: G2: 5% G ≥ 3: 0%	Prevalence at 6 months G2: 14% G3: 0%	Prevalence at 6 months G2: 4% G3: 0%	NR
Fuller et al., 2018 [28] Fuller et al., 2022 [29]	259 pts LR = 53% IR = 57%	Phase II prospective trial	CyberKnife	38 Gy / 4fx	D_max_ < 45.6 Gy (131.3 Gy _EQD2_) D_10%_ < 41.8 Gy (112.4 Gy _EQD2_) D_50%_ < 39.9 Gy (103.6 Gy _EQD2_)	No	5.5 years	CTCAE	G2: 35.1% G3: 1.1%	G2: 6.9% G3: 0%	Cumulative incidence G ≥ 2: 19.2%	Cumulative incidence G2: 4.1%	10-year bRFS: LR: 100% IR-favorable: 84.3% IR-unfavorable: 68.4%
McDonald et al., 2018 [20]	26 pts LR = 33% IR = 77%	Prospective pilot trial	VMAT	36.25 Gy /5fx Dose escalation to 40 Gy /5fx to the DIL	D_max_ ≤ 38.78 Gy (83.5 Gy _EQD2_)	30%	At least 3 months	NR	G2: 7.7% G3: 0%	G2: 7.7% G3: 0%	NR	NR	NR
Brand et al., 2019 [25] Tree et al., 2022 [7]	874 pts (414 pts in the SBRT arm)	Phase III prospective randomized trial (PACE-B)	VMAT IMRT CyberKnife	36.25 Gy and 40 Gy/5fx	V42Gy (95.7 Gy _EQD2_) < 50% V44Gy (103.8 Gy _EQD2_) < 20%	No	24 months	CTCAE	Worst acute toxicity: G ≥ 2: 30.9%	Worst acute toxicity: G ≥ 2: 15.6%	Cumulative incidence G ≥ 2: 32.3%	Cumulative incidence G ≥ 2: 12.5%	NR
Pryor et al., 2019 [27]	135 pts IR = 76% HR = 26%	Phase II prospective trial (PROMETHEUS)	VMAT / IMRT	Prostate: 46 Gy /23fx Followed by a boost: 19-20 Gy /2fx	D_0.1 cc_ < 20.9 Gy (total of 102 Gy _EQD2_) – 22 Gy (total of 107.6 Gy _EQD2_)	36% short-term 18% long-term	24 months	CTCAE	G2: 26.6% G3: 0%	G2: 4.4% G3: 0%	Cumulative incidence G ≥ 2: 24.9% G ≥ 3: 2.2%	Cumulative incidence G ≥ 2: 4.5% G ≥ 3: 2%	NR
Kishan et al*.,* 2023 [36]	156 pts IR = 61% HR = 23% vHR = 9% N + = 7%	Prospective randomized phase III trial (MIRAGE)	MR-guided vs CT-guided SBRT	40 Gy /5fx	Dmax < 42 Gy (95.7 Gy _EQD2_)	CT-arm: 74% MRgRT: 62%	At least 3 months	CTCAE	MRgRT: G ≥ 2: 24.4% CT-guided: G ≥ 2: 43.4%	MRgRT: G ≥ 2: 0% CT-guided: G ≥ 2: 10.5%	NR	NR	NR

*RT* radiation therapy, *GU* genitourinary, *GI* gastrointestinal, *VMAT* volumetric arc therapy, *pts* patients, *LR* low-risk, *IR* intermediate-risk, *HR* high-risk, *NR* non reported, *DIL* dominant intraprostatic lesion, *IMRT* intensity-modulated radiation therapy, *G* grade, *bRFS*biochemical relapse-free survival

Other trials implemented a urethra-steering technique for a whole prostate gland SBRT irradiation. Tree et al*.* recently reported the 2-year toxicity results of patients randomized in the PACE-B phase III clinical trial comparing a 5-fraction SBRT schedule versus moderate or standard fractionation [7]. For the 5-fraction schedule, the constraints to the urethra were optional, with a V_44Gy_ < 20% amended successively in favor of a V_42Gy_ < 50%. The 2-year cumulative incidence of grade ≥ 2 GU toxicity was acceptable, raising to 32.3%. The most frequent grade ≥ 2 GU toxicity was an increased urinary frequency, peaking at 15 months and observed in 10% of patients. Worst acute grade ≥ 2 GU toxicity was reported earlier and raised up to 30.9% of men with a peak 2 weeks after the start of SBRT [25]. Kishan et al*.* recently reported the early toxicity results of the randomized phase III MIRAGE trial, comparing a magnetic resonance (MR)-guided with a computed tomography (CT)-guided prostate SBRT [26]. The clinical target volume was expanded by 4 mm in case of CT-guided SBRT and 2 mm for MR-SBRT and the delivered dose was 40 Gy in 5 fractions, including an additional boost on the DIL or pelvic lymph node radiotherapy. The maximal dose delivered to the urethra was limited to 42 Gy (95.7 Gy EQD2). MR-based SBRT with reduced margins enabled a significant reduction of both acute grade ≥ 2 GU and GI toxicities compared to a CT-based SBRT (24.4% *vs* 43.4%, p = 0.01 and 0% *vs* 10.5%, p = 0.03). Of note, the maximal dose delivered to the urethra was similar between the two arms (41.6 Gy and 41.7 Gy for CT-guidance and MRI-guidance, respectively), while MR-based SBRT was associated with a significant reduction in the volume of bladder receiving 40 Gy (0.3 cc *vs* 0.7 cc, p = 0.001) and 39 Gy (1.9 cc *vs* 3.7 cc, p < 0.001). Pryor et al*.* reported the results of the PROMETHEUS trial, evaluating the use of a high-dose ultra-hypofractionated SBRT boost as dose-escalation strategy for men with either intermediate-risk or high-risk PCa [27]. Prostate SBRT boost consisted of either 19 Gy or 20 Gy in two fractions, followed by a prostate radiotherapy at a dose of 46 Gy in 23 fractions. The D_0.1 cc_ delivered to the urethra was limited to < 110% of the total SBRT dose. Acute and cumulative rates of grade ≥ 2 GU toxicities were reported in 26.6% and 27.1%, respectively, with a peak observed at 18 months. Fuller et al*.* reported the outcomes of 259 low- and intermediate-risk PCa patients treated within a phase II trial of “high-dose rate (HDR)-like” SBRT [28, 29]. The prescribed dose was 38 Gy in 4 fractions, with planning target volume receiving at least 150% of the prescription dose. The maximal dose constraint imposed on the urethra (D_max_ < 131.3 GyEQD2) was significantly higher than what was previously reported [7, 21, 23]. A deterioration of QoL scores was noted at one month, with the appearance of obstructive complaints and weak stream in 15% and 8% of patients, respectively, returning to baseline by 6 months. A 36.2% and 19.2% rate both acute of late grade ≥ 2 GU toxicity were reported, including up to 10% of the patients reporting the use of incontinence pads after 5 years of follow-up. Of note, one patient required a total cysto-prostatectomy for a grade 4 cysto-urethritis. While this study recruited a 4% rate of patients with prior transurethral resection of the prostate (TURP), authors suggest a cautious patient selection when high biologically effective dose are delivered to the whole prostate gland. Excellent biochemical control was demonstrated in men with low-risk PCa, with a 10-year biochemical relapse free-survival (bRFS) of 100%. However, the 10-year bRFS reached only 68.4% for unfavorable intermediate-risk PCa patients, probably attributable to the lack of ADT prescription.

## Urethra dose-reduction SBRT

A urethra dose-reduction strategy was tested in two prospective phase II SBRT trials [30, 31] (Table 2). While a total dose of 36.25 Gy in 5 fractions was prescribed to the whole prostate gland, a dose reduction to 32.5 Gy was delivered to a 2-mm planning organ-at-risk volume (PRV) generated around the urethra. This dose reduction was adopted as best dose compromise in an attempt to minimize GU toxicity while maintaining an acceptable tumor control to the possible microscopic periurethral disease (74 Gy _EQD2_, α/β = 1.5 Gy). In a phase II multicenter randomized clinical trial, Zilli et al*.* tested this optimization strategy using two different schedules, delivered every-other-day (EOD) or once-a-week (QW) [31, 32]. Among the 165 patients treated, mostly diagnosed with either low- or intermediate-risk PCa, acute toxicity was mild or absent, with no differences between arms. With a follow-up of more than 70 months, the incidence of CTCAE grade 2 GU toxicity was below 10% for both arms, respectively, corresponding to a 5-year grade 2 or greater GU toxicity-free survival of 75.9% and 76.1% for patients treated EOD versus QW, respectively (P = 0.945). Together with a minimal impact on QoL, oncological outcomes were encouraging, with a 5-year bRFS exceeding 90% for both fractionations. Of note, the trial reported dosimetry protocol deviations in 31% of the cases, consisting mainly of underdosing of urethral PRV (12% of the patients with D_98%_ < 30.2 Gy), particularly when using an IMRT technique [33]. Using the same fractionation schedule delivered with adaptive Magnetic Resonance-guided Radiotherapy (MRgRT), Bruynzeel et al*.* reported a 19.8% of grade 2 CTCAE GU toxicity at the end of SBRT, decreasing to 7.9% at 6-weeks and remaining between 3.1% and 5.1% thereafter [30, 34]. This trial recruited a majority of patients with high-risk PCa. To date, no-long-term oncological results are available.

**Table 2:** Toxicity and oncological outcomes after urethra dose-reduction protocols (prescription of a lower dose on the whole urethra, or with maximal doses delivered to the urethra lower than the dose of prescription to the prostate gland)

Author	Patients	Study design	RT technique	RT dose	Urethral dose prescription	Dose constraints (EQD2, α/β = 3 Gy)	ADT	Median follow-up	Toxicity scale	Acute GU toxicity	Acute GI toxicity	Late GU toxicity	Late GI toxicity	Oncological outcomes
Bruynzeel et al*.*, 2020 [30] Tetar et al., 2021 [34]	101 pts LR = 4% IR = 37% HR = 59%	Phase II prospective trial	MRgRT	36.25 Gy / 5 fx	32.5 Gy /5fx	D_2%_ < 34.8 Gy (69.2 Gy _EQD2_)	41% short-term 41% long-term	At least 3 months	CTCAE	G ≥ 2: 19.8%	G ≥ 2: 3%	G2: 3.1–5.1%	0%	2-year bRFS: 96.7%
Greco et al., 2022 [37]	444 pts LR = 4.1% IR = 84% HR = 11.9%	Phase II prospective trial	VMAT	45 Gy /5fx	None	D_1cc_ < 36 Gy (73.4 Gy _EQD2_)	36%	58 months	RTOG	G2: 6.8% G3: 0%	G2: 0.5% G3: 0%	Cumulative incidence: G ≥ 2: 5.3%	Cumulative: G ≥ 2: 1.1%	7-year bRFS: 86.2%
Greco et al., 2021 [38]	30 pts IR = 100%	Phase II prospective randomized trial (PROSINT)	VMAT	45 Gy /5fx	None	D_max_ < 42.75 Gy (98.7 Gy _EQD2_) D_1cc_ < 36 Gy (73.4 Gy _EQD2_)	No	48 months	RTOG	G ≥ 2: 0%	G ≥ 2: 0%	Cumulative incidence: G2: 17%	G ≥ 2: 0%	4-year bRFS: 85.7%
				24 Gy /1fx		D_max_ < 22.8 Gy (117.6 Gy _EQD2_) D_1cc_ < 19.2 Gy (85.2 Gy _EQD2_)				G ≥ 2: 0%	G ≥ 2: 0%	Cumulative incidence G2: 11.4% Urethral strictures: 6.6%	G ≥ 2: 0%	4-year bRFS: 77.1%
Magli et al., 2021 [36]	59 pts	Phase II prospective trial	IMRT	40 Gy / 3 fx	33 Gy /3fx	D_0.1 cc_ < 33 Gy (92.4 Gy _EQD2_)	3 months if prostate size > 80cm^3^	At least 12 months	CTCAE	G ≥ 2: 13.8%	G2: 8.5%	Prevalence at 12 months: G2: 0%	G2: 0%	NR
Parsai et al., 2020 [35]	35 pts LR = 9% IR = 40% HR = 51%	Prospective pilot study	VMAT	50 Gy /5fx 36.25 Gy /5fx to HDAZ	36.25 Gy /5fx	D_max_ < 50 Gy (130 Gy _EQD2_) D_1cc_ < 45 Gy (108 Gy _EQD2_)	No: 45% Short-term: 50% Long-term: 5%	46 months	CTCAE	G2: 19.4% G4: 2.9%	G2: 0% G4: 2.9%	12-months incidence: G2: 25% G4: 2.9%	12-months incidence: G2: 5.6% G4: 2.9%	3-year bRFS: 88% LR: 100% IR: 89.5%-100% HR: 82.3%
Zilli et al., 2019 [39]	6 pts	Phase I prospective trial (ONE SHOT)	VMAT	19 Gy /1fx	17 Gy /1fx	D_2%_ < 18.2 Gy (77.1 Gy _EQD2_)	No	At least 3 months	CTCAE	G2: 33% G ≥ 3: 0%	G2: 0%	NR	NR	NR
Zilli et al., 2020 [32] Zilli et al., 2023 [31]	170 pts LR = 22% IR = 64% HR = 14%	Phase II randomized trial (once a week vs every other day)	IMRT / VMAT	36.25 Gy /5fx	32.5 Gy /5fx	D_2%_ < 34.8 Gy (69.2 Gy _EQD2_)	45%	77/78 months	CTCAE	Worst G2: 17/19%	Worst G2: 2/0%	Cumulative: G2: 21.6% G3: 0.6% Incidence: G2 8.3% and 7.3% (5-yr)	Cumulative: G2: 9.3% G3: 0% Incidence: G2 < 2% (5- yr)	5-year bRFS: 92.2% (every other day) – 93% (once a week)

*RT* radiation therapy, *GU* genitourinary, *GI* gastrointestinal, *MRgRT* magnetic resonance guided radiation therapy, *NR* non reported, *bRFS* biochemical relapse-free survival, *pts* patients, *LR* low-risk, *IR* intermediate-risk, *HR* high-risk, *IMRT* intensity modulated radiation therapy, *HDAZ* high dose avoidance zone, *NS* non specified, *G* grade, *bRFS* biochemical relapse-free survival

Parsai et al. further implemented urethra dose-reduction within a “high-dose avoidance zones” (HDAZ) protocol, defined as a 3-mm expansion around rectum, urethra, and bladder [35]. A dose of 50 Gy in 5 fractions was prescribed to the target volume, with a dose reduction as low as 36.25 Gy on the prostate gland in close proximity with organs at risk. Urethral D_max_ and D_1cc_ were deemed to be less than 130 GyEQD2 and 108GyEQD2, respectively. At a median follow-up of 46 months, a 19.4% and 25% rate of acute and late grade 2 GU toxicity were observed, respectively, consisting mostly of urinary irritation and obstructive symptoms. One patient developed Fournier gangrene after implantation of radiofrequency transponders, requiring multiple surgeries for debridement. For the whole cohort, the 3-year bRFS was 88%, while the same rate decreased to 82.3% in patients with high-risk disease. In a dose escalated phase II trial, Magli et al*.* tested a three fractions SBRT schedule up to 40 Gy, with a dose reduction to 33 Gy to the urethral PRV [36]. Acute grade ≥ 2 toxicity was reported in 13.8% of the patients, consisting mostly in irritative symptoms rapidly improving 1 month after treatment end. At 1 year, no patient experienced persistent grade 2 GU toxicity. Greco et al*.* recently reported the outcomes of 444 men treated within a phase II trial at a dose of 45 Gy in 5 fractions on the prostate gland [37]. Most patients were diagnosed with intermediate-risk PCa (84%), with only a small proportion of men presenting with high-risk PCa (11.9%). The maximal dose delivered to the urethra was limited to 36 Gy (73.4 Gy _EQD2_). A Foley catheter loaded with 3 electromagnetic transponders was used for both urethra visualization and tracking. Only 6.8% of the patients experienced grade 2 toxicity, with 4 cases of acute retention requiring catheterization. Excellent oncological outcomes were demonstrated in the whole population with a 7-year bRFS of 86.2%, yet reaching only 73.5% in the high-risk population. Of the 34 patients with positive positron emission tomography/computed tomography (PET/CT) findings at relapse, 73.5% showed evidence of intraprostatic relapse at the site of the pre-treatment DIL.

Two studies assessed the safety and efficacy of a single-dose SBRT for men with localized PCa. Using the same tracking approach, Greco et al*.* randomized in the PROSINT trial 30 men to receive either 45 Gy in 5 fractions or 24 Gy in one fraction [38]. In the single-dose arm, urethral D_max_ and D_1cc_ were respectively constraints to 22.8 Gy (117.6 GyEQD2) and 19.2 Gy (85.2 GyEQD2). While no patient experienced grade 2 GU toxicity in the acute setting, one patient out of fifteen experienced urethral stricture at 30 months of follow-up. The 4-year bRFS reached only 75% and 64% for men with unfavorable intermediate-risk disease in the 5 fraction and single-fraction arm, respectively. Zilli et al*.* also explored in a single-arm multicenter phase I/II trial the safety and efficacy of a single-fraction SBRT for men presenting with low- or intermediate-risk PCa [39]. In the “ONE SHOT” trial, the prostate gland was planned to receive a dose of 19 Gy, while a dose-reduction at 17 Gy was performed to the urethral PRV. In the phase I, fifty percent of men reported grade 2 toxicity at 1 week after treatment, returning to baseline at 12 weeks. No grade 3 toxicity was reported with a minimal dosimetric impact of intrafraction prostate motion by using real-time electromagnetic tracking combined with beam gating [40].

## Discussion

The urethra can be considered as a tubular “serial” structure, and most US-SBRT trials investigated the association between the maximum doses to the urethra and the onset of late GU toxicity. The moderate hypofractionation FLAME trial (77 Gy in 35 fractions with or without a 95 Gy dose escalation on the DIL) reported an exponential dose-toxicity relationship, with a strong correlation with urethra D_0.1 cc_ metrics [41]. The authors proposed to implement a dose constraint of 80 Gy when SIB optimization is performed (D_0.1 cc_ ≤ 91.2GyEQD2). These constraints are broadly similar to those published by Zhang et al. for a 38 Gy/4 fractions HDR-like SBRT schedule, advising in favour of the implementation of a urethral maximal dose constraint of 38/42 Gy (D_max_ < 80.6 – 95.8 GyEQD2) [42]. More recently, the meta-analysis of 23 SBRT prospective trials led by Leeman et al*.* demonstrated a significant association between urethral doses and onset of late GU toxicity, with each increase in 1 Gy in maximal urethral doses corresponding to a 0.8% and 1% increase in acute and late grade ≥ 2 GU toxicity [16]. According to this model, a maximal urethral dose of 100 GyEQD2 would result in a 10% probability to experience late grade ≥ 2 GU toxicity. The vast majority of the urethra-sparing trials have imposed urethral D_max_ below this threshold within their protocol [20, 21, 24, 30, 31, 36, 36, 37, 43] and demonstrated the efficiency of this approach in reducing high-grade late toxicities. In trials restricting urethra D_max_ to 90 GyEQD2, the rates of late grade 2 toxicity ranged from 12.1% [20] to 14% [21], with no report of urethral stenosis. On the other hand, trials imposing less stringent urethral constraints (D_max_ > 100GyEQD2) [7, 25, 27–29, 35, 38] attested significantly higher rates of late grade 2 GU toxicity, with cumulative incidences ranging from 17 to 32%, together with the onset of severe toxicity consisting either in urethral strictures [38] or urethritis requiring cysto-prostatectomy [28]. Although both US-SBRT techniques have been associated with promising mitigation of GU toxicity, a strict comparison between the two optimization strategies remains difficult. While dose reduction strategies with strict urethral dose constraints may represent a valid option when the prostate is treated with a homogeneous dose and the dominant tumor is not located closely to the transition zone, urethra-steering may be the technique of choice when dose-escalation on the DIL is performed.

While most SBRT studies assessed the impact of dose-volume parameters delivered to the intraprostatic urethra, the dose delivered to other urethral segments has also been suggested to be associated with the late onset of GU toxicity. Several retrospective series with HDR BT suggested the bulbo-membranous urethra to be the most radiosensitive segment, after reporting this portion as the one most frequently affected by stenosis [14, 44]. Additionally, Mohammed et al*.* showed a significant association between the risk to develop a urethral stricture and the maximal doses delivered to the bulbo-membranous urethra [45]. Low-to-intermediate radiotherapy doses delivered to the bulbo-membranous urethra were also associated with the occurrence of late onset dysuria in a voxel-based analysis performed in patients treated within the RADAR and CHHiP trials [46]. The development of predictive pixel and voxel approaches also led to the development of the hypothesis of a heterogeneous intra-organ radiosensitivity. Mylona et al*.* recently identified the volume of bladder trigone receiving > 72 Gy as a predictor of acute urinary retention [47]. Also using a voxel-based analysis, Improta et al. found an association between the dose delivered to the bladder trigone and the risk of acute GU toxicity [48]. Last but not least, Ghadjar et al*.* demonstrated an association between various trigone dose-parameters and the occurrence of overall grade ≥ 2 GU toxicity and late obstructive voiding symptoms [49]. Future trials delineating the different urinary sub-structures separately and investigating dose–volume relationships in these regions are awaited to further characterize their dose sensitivity.

Beyond the dose parameters delivered to urinary structures, the occurrence of late GU toxicity is known to be multifactorial. While transurethral resection of the prostate (TURP) has long been associated with the onset of urethral strictures after BT [14], the impact of surgical treatments of benign prostatic hyperplasia has been poorly studied in SBRT trials. In a retrospective study including 47 patients treated with SBRT, Pepin et al*.* reported late grade 2 and 3 GU toxicity raising up to 48.9% and 6.4% of the patients, respectively, consisting mostly in haematuria in relation to necrosis occurring in the bladder neck or TURP defect [50]. Huck et al*.* also reported late grade 2 and 3 GU toxicity in 33% and 17% of patients with a previous history of surgical treatment for benign prostatic hyperplasia, occurring more frequently in patients with prior adenomectomy, multiple TURP and/or large volumes of the intraprostatic resection cavity [51]. Up to 42% of the patients experienced at least one episode of hematuria. Prostate volume (> 50–60 cc) has also been suggested to be a predictor of both acute and late GU toxicity, without any threshold being formally identified [52, 53]. In men presenting with large prostate (> 50 cc), a late urinary flare consisting mostly in dysuria and retention has been observed up to two years after prostate SBRT, yet with no impact on quality of life [54]. In a population of patient with a prostate size > 100 cc, Haas et al*.* also demonstrated a transient decline in EPIC scores at 1 and 3 months after SBRT, resolving by 1 year after treatment completion [55]. Although discrepancies still exist between studies, prostate size may not be one of the strongest determinants of urinary toxicity after SBRT.

The development of urethra-sparing techniques for PCa has initially been discouraged due to the report of unusually high rates of biochemical failure within the first phase II trial led by Vainshtein et al*.* [56]*.* More recent urethra-dose reduction trials reported encouraging results in terms of biochemical control, despite restrictions due to a short follow-up. Zilli et al*.* recently reported a 5-year bRFS of 92.2% with 36.25 Gy in 5 fractions schedule performed EOD, in a population of patients mostly represented by low-risk or intermediate-risk PCa [31]. Greco et al*.* demonstrated a 7-year bRFS of 86.2% with dose-escalation up to 45 Gy, with a cumulative incidence rate of PSA failure of 2%, 16.6%, and 27.2% in the low- and favorable intermediate-, unfavorable intermediate-risk, and high-risk groups, respectively [37]. Excellent oncological results were also demonstrated by Fuller et al., with a 10-year bRFS reaching 100% and 84.3% in the low-risk and the favorable intermediate-risk cohort, respectively, with only 3 reports of biopsy-proven local recurrence [28]. While data support the safety of implementation of US-SBRT for low- to intermediate-risk PCa, data remain scarce with regard to high-risk PCa. Parsai et al. reported a 3-year bRFS of 82.3% in men presenting with high-risk disease, which compares favorably with a pooled meta-analysis published by King et al*.* showing a 5-year bRFS of 81% in this population of patients [57]. None of these studies required a minimum distance between the urethra and the intra-prostatic tumor, and to date only Cloitre et al*.* deemed a 3-mm minimal distance between the tumor and the urethra to safely adopt urethra-sparing techniques. [23].

Several ongoing SBRT trials are implementing urethra-sparing techniques to mitigate long-term GU toxicity (Table 3). Precise definition of the urethra represents one the major limitations to the implementation of this technique in clinical practice. Although use of a Foley catheter is the standard technique used to define the urethra [58], the invasive nature of this technique and the risk of plan uncertainties due urethral displacements [59] limit its widespread application in clinical practice. The use of MRI with dedicated sequences and automatic segmentation based on artificial intelligence (AI) are promising tools increasingly used to improve the accuracy in the definition of the urethra. Integration of these technologies into modern MRI-linacs, makes MR-guided SBRT an appealing treatment option to treat PCa patients. The definition of the urethra on dedicated MRI sequences, the use of adaptive treatment delivery with reduced PTV margins [26], and the possibility of optimization on other structures involved in GU toxicity (trigone, bladder neck, bulbous and membranous portions of the urethra) constitute the main advantage of this technology compared to standard CT-guided SBRT techniques.

**Table 3:** Ongoing prostate SBRT trials implementing urethra-sparing techniques

Trial	Design	Technique	Dose delivered to the target volume	Dose delivered to the urethra	Primary outcome
NCT04896801 (Proseven)	Single arm prospective trial	MRgRT	PTV: 36 Gy/5fx (90% isodose line) Prostate gland: 40 Gy /5fx DIL: 42 Gy/5fx	V40Gy: < 1 cc	Acute toxicity (CTCAE and RTOG)
NCT05936736 (PRO-FAST)	Single arm prospective trial	NR	PTV: 24 Gy /1fx	NR	Acute toxicity (CTCAE)
NCT05919524 (SAFO)	Single arm prospective trial	NR	PTV: 36.25 Gy /5fx DIL: 50 Gy /5fx	NR	bRFS Local PFS
NCT05668351 (SUPR-SABR)	Single arm prospective phase II trial	NR	PTV: 40 Gy /5fx	Dmax: 36.25 Gy	Toxicity (EPIC score)
NCT05804318 (ARTIA- prostate)	Single arm prospective trial	Adaptive RT	PTV: 40 Gy /5fx	Prescription dose: 35-36 Gy/5fx	Acute toxicity (EPIC)
NCT02470897	Randomized prospective trial	IMRT	PTV: 37.5 Gy or 40 Gy /5fx	NR	Acute and late toxicity bRFS

*PTV* planning target volume, *DIL* dominant intraprostatic lesion, *MRgRT* magnetic resonance-guided radiotherapy, *fx* fractions, *bRFS* biochemical relapse-free survival, *PFS* progression-free survival, *RT* radiotherapy

This systematic review has several limitations. First, a comprehensive overview of studies performing urethra-sparing radiotherapy remains difficult to be conducted, due to the lack in some cases of information on urethra-sparing procedures. Toxicity evaluation was also heterogeneous among studies, including use of different grading scales (either RTOG and/or CTCAE). Also, protocol violations or “real-life” doses delivered to the urethra were not reported in most trials, which represents a limitation in the interpretation of toxicity outcomes. Moreover, delineation of urethra has been performed using either a Foley catheter [31, 35, 36, 38, 39, 43] or a co-registration with the diagnostic MRI [23, 30]. Last but not least, some studies implemented a 2–3-mm PRV margin around the urethra [21, 30, 31, 35, 36, 39], while other did not [23, 29, 38], leading to a large variation in the sparing and treatment optimization of this structure.

## Conclusions

In patients with localized prostate cancer, US-SBRT techniques limiting maximum doses to urethra below a 90-GyEQD2 (α/β = 3 Gy) threshold represent a promising strategy to mitigate acute and long-term grade ≥ 2 GU toxicity, while maintaining at the same time acceptable rates of local disease control. Dose-reduction to urethra below 70 Gy_EQD2_ (α/β = 3 Gy) may enable a further reduction in long-term GU toxicity in selected patients with no tumour in the transition zone. A better understanding of the clinical factors and anatomical substructures involved in the development of urinary toxicity, as well as the development and use of adapted dose constraints, will help to further reduce the long-term GU toxicity of patients undergoing SBRT for prostate cancer.
